# Bird-Like Anatomy, Posture, and Behavior Revealed by an Early Jurassic Theropod Dinosaur Resting Trace

**DOI:** 10.1371/journal.pone.0004591

**Published:** 2009-03-04

**Authors:** Andrew R. C. Milner, Jerald D. Harris, Martin G. Lockley, James I. Kirkland, Neffra A. Matthews

**Affiliations:** 1 St. George Dinosaur Discovery Site at Johnson Farm, St. George, Utah, United States of America; 2 Dixie State College, St. George, Utah, United States of America; 3 Dinosaur Tracks Museum, University of Colorado at Denver, Denver, Colorado, United States of America; 4 Utah Geological Survey, Salt Lake City, Utah, United States of America; 5 National Operations Center, USDOI-Bureau of Land Management, Denver, Colorado, United States of America; University of Utah, United States of America

## Abstract

**Background:**

Fossil tracks made by non-avian theropod dinosaurs commonly reflect the habitual bipedal stance retained in living birds. Only rarely-captured behaviors, such as crouching, might create impressions made by the hands. Such tracks provide valuable information concerning the often poorly understood functional morphology of the early theropod forelimb.

**Methodology/Principal Findings:**

Here we describe a well-preserved theropod trackway in a Lower Jurassic (∼198 million-year-old) lacustrine beach sandstone in the Whitmore Point Member of the Moenave Formation in southwestern Utah. The trackway consists of prints of typical morphology, intermittent tail drags and, unusually, traces made by the animal resting on the substrate in a posture very similar to modern birds. The resting trace includes symmetrical pes impressions and well-defined impressions made by both hands, the tail, and the ischial callosity.

**Conclusions/Significance:**

The manus impressions corroborate that early theropods, like later birds, held their palms facing medially, in contrast to manus prints previously attributed to theropods that have forward-pointing digits. Both the symmetrical resting posture and the medially-facing palms therefore evolved by the Early Jurassic, much earlier in the theropod lineage than previously recognized, and may characterize all theropods.

## Introduction

Theropod dinosaurs, exemplified by such animals as *Dilophosaurus*, *Allosaurus*, *Velociraptor*, and *Tyrannosaurus*, are among the most successful dinosaurian clades, and the only one with representatives – namely, birds – known to survive the end-Cretaceous extinction event. Theropods skeletal fossils are also components of some of the oldest known (Late Triassic and Early Jurassic) terrestrial faunas, though many aspects of the anatomy of these early taxa are poorly known compared to their younger counterparts.

Late Triassic-Early Jurassic dinosaur ichnites (trace fossils), are dominated by ichnotaxa attributed to non-avian theropods. All known theropods are perceived as obligate bipeds [1]; no known theropod habitually adopted a quadrupedal posture for locomotion [2]. Theropod trackways therefore do not typically exhibit hand imprints. Only when the trunk was lowered toward a substrate, as in a crouched posture, could the hands potentially create impressions.

Most previously reported dinosaurian crouching (resting) traces, such as those of the ichnotaxon *Anomoepus*, have usually been attributed to bipedal, herbivorous, ornithischian dinosaurs [3]–[7]. Traces interpreted as having been made by crouching or resting theropods are exceptionally rare: only six examples have been reported based on adequate information. Four of these lack manus impressions, including a briefly-described exemplar from China and a specimen pertaining to the small theropod ichnotaxon *Grallator* [5]. The remaining two, also referable to *Grallator*, have associated but faint, amorphous hand imprints [5], [8]. In two other described theropod trackways, not made by crouching animals, purported hand traces are faint and lack detail [5], [9], [10].

**Figure 1:**
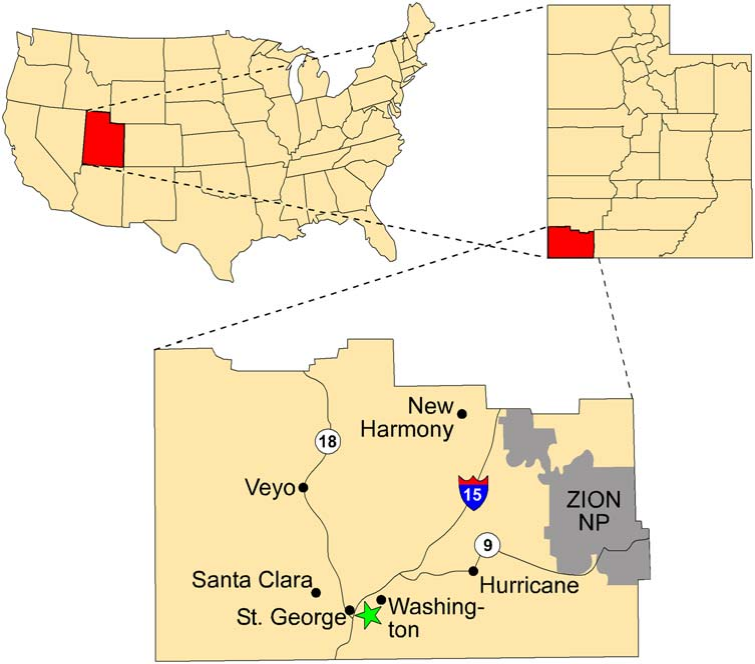
Location of the St. George Dinosaur Discovery Site at Johnson Farm (SGDS) (green star) in Washington County, southwestern Utah. The site and others within the 1 km^2^ mentioned in the text are within the boundaries of the City of St. George.

Here we describe a well-preserved crouching theropod trace from a lacustrine beach sandstone of the Lower Jurassic (Hettangian, ∼198 Ma) Whitmore Point Member of the Moenave Formation in southwestern Utah (Figure 1) [11]. The trace is part of a longer, hind foot-only trackway (SGDS.18.T1) that also includes intermittent tail drags. The crouching trace was registered when the animal rested on the substrate in a posture very similar to modern birds; the traces include well-defined impressions made by both pedes, both hands, the tail, and the ischial callosity. This trace constitutes evidence that an Early Jurassic theropod expressed two bird-like features: anatomical restriction to a palms-medial manual posture, and symmetrical leg positions while resting.

### Stratigraphic and Paleoecological Setting

**Figure 2:**
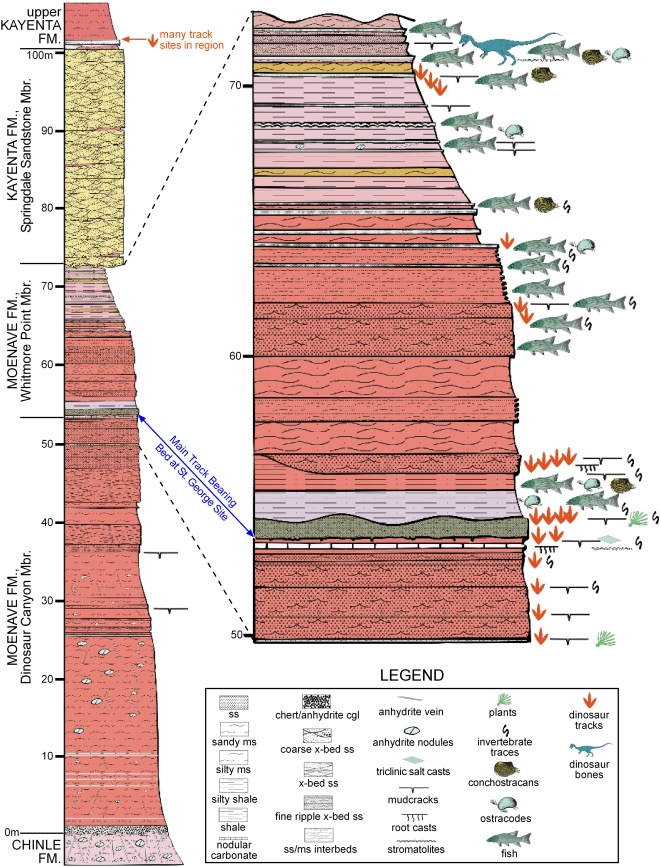
Stratigraphic section of the Moenave Formation at the St. George Dinosaur Discovery Site at Johnson Farm. Resting trace and trackway SGDS.18.T1 is in the “Top Surface” of the Main Track-Bearing Sandstone Bed (indicated by the blue arrow) in the Whitmore Point Member of the Moenave Formation.

**Figure 3:**
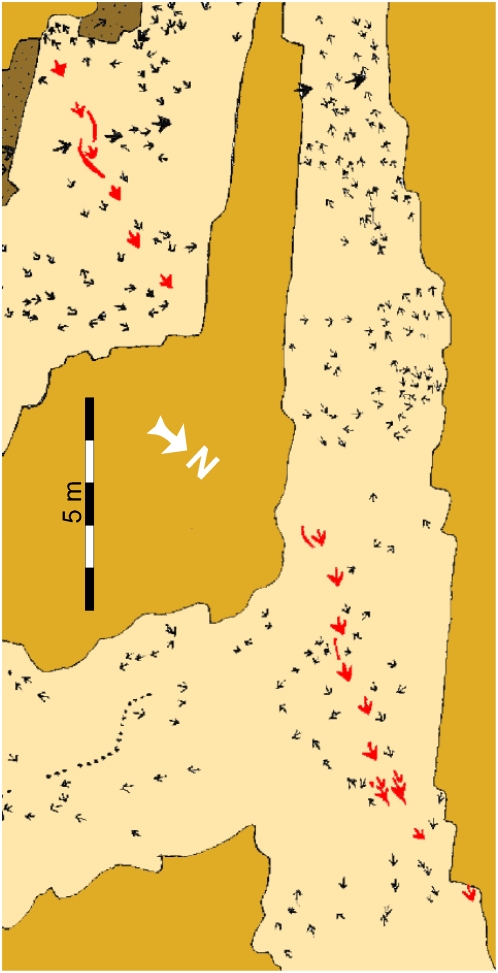
Schematic map of the “Top Surface” tracksite (SGDS.18). Beige shaded areas represent the “Top Surface” of the Main Track-bearing Sandstone Bed; gold shaded areas are unexcavated; brown areas represent areas removed after mapping to examine lower horizons. The *Eubrontes* trackway that includes the crouching trace is highlighted in red.

**Figure 4:**
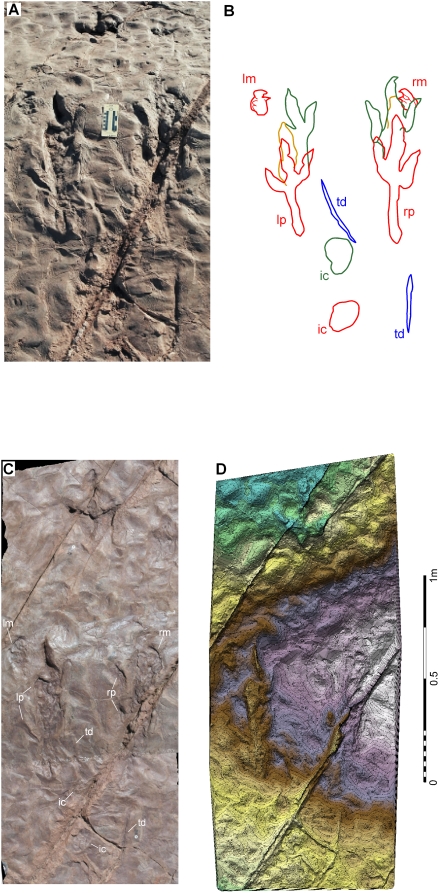
*Eubrontes* trackway with resting trace (SGDS.18.T1) in the Whitmore Point Member of the Moenave Formation, St. George, Utah. A, Overhead, slightly oblique angle photograph of SGDS.18.T1 resting trace. Note normal *Eubrontes* track cranial to resting traces (top center) made by track maker during first step upon getting up. Scale bar equals 10 cm. B, Schematic of SGDS.18.T1 to scale with A: first resting traces (manus, pes, and ischial callosity) in red, second (shuffling, pes only) traces in gold, final resting traces (pes and ischial callosity) in green, and tail drag marks made as track maker moved off in blue. Note long metatarsal (“heel”) impressions on pes prints. C, Direct overhead photograph and D, computerized photogrammetry with 5 mm contour lines of *Eubrontes* trace SGDS.18.T1. Color banding reflects topography (blue-green = lowest, purple-white = highest); a portion of the berm on which the track maker crouched is discernible. Abbreviations: ic = ischial callosity, lm = left manus, lp = left pes, rm = right manus, rp = right pes, td = tail drag marks.

**Figure 5:**
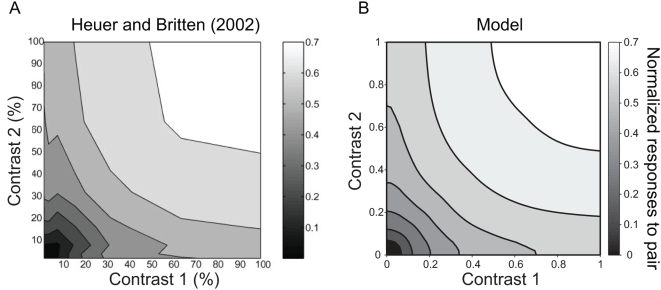
Stereophotographs of SGDS.18.T1 crouching trace. Elevation exaggerated to emphasize individual tracks. Placards on surface are markers used in generation of photogrammetric image in Figure 4D.

Twenty-five track-bearing horizons contained within a small area (1 km^2^) in St. George, Utah, contain a diverse, theropod-dominated ichnofauna. The most fossiliferous and diverse surface (Figure 2) is preserved within the St. George Dinosaur Discovery Site at Johnson Farm (SGDS) museum [12]. Mudflat, shoreline, and periodically submerged surfaces coincide on the same bedding plane as evidenced by mud cracks, ripple marks (current, symmetrical, wind-driven, interference, and wave-formed), erosive mega-ripples, load and flute casts, rill and tool marks of various sizes, raindrop impressions, and invertebrate and vertebrate ichnites. This suite of sedimentary features formed on a beach or shoal along the shores of an Early Jurassic freshwater body (Lake Dixie) that underwent seasonal regressive-transgressive fluctuations [11]. The majority of theropod trackways on this surface trend north-south, paralleling the paleoshoreline. The 22.3 m long SGDS.18.T1 trackway (Figure 3) includes the unique crouching traces (Figures 4, 5). The non-crouching pes prints in the trackway conform to the large theropod ichnotaxon *Eubrontes* (Table 1; see below) for which resting traces and tail drags are extremely rare.

**Table 1:** Measurements (in cm; ° as noted) of SGDS.18.T1 *Eubrontes* trackway and corresponding tail drag marks.

Track #	L/R	TL	TTL	TW	MTD	I–II	I–III	I–IV	II–IV	II–III	III–IV	PA	P	S
T1.1	R	34	34	28.5	(3.5)	–	–	–	65°	33°	36°	–	–	–
T1.2	L	–	–	(27)	–	–	–	–	–	–	–	–	–	–
T1.3	R	–	–	–	–	–	–	–	–	–	–	–	–	–
T1.4–1.9	associated with resting traces – see Table 2													
T1.10	L	(38)	(42.5)	(29)	(4.5)	91°	139°	(187°)	81°	45°	48°	–	–	–
T1.11	R	39	39	28	3.5	–	–	–	61°	36°	32°	166°_10–12_	101_10–11_	200_10–12_
T1.12	L	(36)	(36)	(26)	(3.5)	60°	110°	160°	82°	49°	36°	160°_11–13_	107_11–12_	200_11–13_
T1.13	R	(35)	–	(24–30)	(3.5)	–	–	–	–	–	–	175°_12–14_	102_12–13_	(195)_12–14_
T1.14	L	(25–43)	–	(25)	(2.5)	–	–	–	–	–	–	157°_13–15_	(100)_13–14_	(232)_13–15_
T1.15	R	(37–43)	–	(25–32)	(5–6)	–	–	–	(82°)	(38°)	(50°)	167°_14–16_	(145)_14–15_	(268)_14–16_
T1.16	L	(35)	45.5	27	6.5	79°	155°	203°	102°	52°	53°	158°_15–17_	145_15–16_	265_15–17_
T1.17	R	(38)	–	(26–30)	(3.5)	–	–	–	–	–	–	150°_16–18_	130_16–17_	248_16–18_
T1.18	L	42	44	27	(1.5–2)	94°	132°	(174°)	77°	41°	46°	156°_17–19_	140_17–18_	247_17–19_
T1.19	R	37	(42)	30	(1)	–	–	–	70°	32°	42°	156°_18–20_	127_18–19_	228_18–20_
T1.20	L	(41)	43	(30)	(2–2.5)	68°	151°	186°	72°	32°	37°	157°_19–21_	116_19–20_	226_19–21_
T1.21	R	(35)	–	27	–	(110°)	(143°)	(191°)	(61°)	(36°)	(25°)	166°_20–22_	119_20–21_	232_20–22_
T1.22	L	(45)	–	(33)	(3)	–	–	–	82°	(42°)	(43°)	167°_21–23_	125_21–22_	243_21–23_
T1.23*	R	(32)	(32)	(28)	–	–	–	–	(59°)	(30°)	(34°)	179°_22–24_	(122)_22–23_	236_22–24_
T1.24*	L	(28)	(25)	–	–	–	–	–	–	–	–	178°_23–25_	(103)_23–24_	205_23–25_
T1.25*	R	32	(40)	(27)	–	(132°)	(168°)	(202°)	(72°)	(34°)	(29°)	176°_24–26_	104_24–25_	196_24–26_
T1.26*	L	(30)	(42)	(24)	(6)	–	–	–	(59°)	(27°)	(34°)	–	110_25–26_	–

Roman numerals = divarication angles between indicated pedal digits, L/R = left/right, MTD = maximum track depth, P = pace or step length, PA = pace angulation, S = stride length, TL = track length (excluding metatarsal impression, if any), TTL = total track length (including metatarsal impression, if any), TW = track width, * = more accurate measurements taken from 2000 M.G. Lockley tracing (CU Denver Tracks Museum Tracing # T472), although tracing provides little data and present track is much more weathered, – = measurement not applicable or unobtainable.

Measurements in parentheses are approximations due to incompleteness, poor preservation of trace, or ambiguity in discerning track margin from surface sedimentary structures; subscripted numbers indicate tracks between which P, PA, and S measurements are given.

Trackway SGDS.18.T1 lies within the basal portion of the Hettangian Whitmore Point Member of the Moenave Formation (basalmost Glen Canyon Group [13]–[15]), approximately 2 m above the underlying Dinosaur Canyon Member (Figure 2). The Dinosaur Canyon Member is dominated by fluvial sandstones and sheet flood deposits laid down along the western edge of Early Jurassic Lake Dixie [15]–[17]. The surface on which this *Eubrontes* trackway is situated (hereafter referred to as the “Top Surface”) is interpreted as an extensive mudflat bordering the western shoreline of Lake Dixie. The “Top Surface” and surrounding horizons are among the lowest of the 25 regional track-bearing horizons, which range stratigraphically from the top of the Dinosaur Canyon Member through the Whitmore Point Member (Figure 2). Theropod footprints are also preserved, albeit less commonly, in the palustrine, fluvial, and, later, eolian settings of the overlying Kayenta Formation [15], [18]–[23].

The Moenave Formation overlies the Chinle Formation (Chinle Group of Lucas [24], but in Utah, group status is not recognized for these same strata). The basal Dinosaur Canyon Member of the Moenave Formation grades gradually eastward from fluvial to eolian facies; on the Colorado Plateau, these eolian deposits, called the Wingate Formation, also overlie the Chinle Formation [25]. The Triassic-Jurassic transition lies in the lower Wingate Formation and thus the lower Moenave Formation [26]–[28], though its precise stratigraphic position remains unknown. The faunas (body fossil and ichnological) of the Church Rock (Rock Point of some authors) Member of the Chinle Formation and the lower Wingate Sandstone are very similar to those of the Dinosaur Canyon Member of the Moenave Formation [28]. The lower Dinosaur Canyon Member thus correlates with the Church Rock Member of the Chinle Formation and the Wingate Sandstone [28] to the east. To the west, in southern Nevada and southeastern California, thinning and unfossiliferous sediments equated with undifferentiated Moenave and Kayenta formations underlie the eolian Aztec Sandstone [29].

Other ichnofossils associated with *Eubrontes* tracks at the SGDS include those of smaller theropods (*Grallator*, ?*Stenonyx*), other large theropods (*Gigandipus*, *Kayentapus*), ornithischians (*Anomoepus*), early crocodylomorphs (*Batrachopus*, *Selenichnus*), probable sphenodontians (*Exocampe*), possible synapsid tracks (?*Brasilichnium*), horseshoe crabs (*Kouphichnium*), insect trails (*Diplichnites*, cf. *Bifurculapes*, *Helminthoidichnites*), invertebrate burrows (*Skolithos*, *Palaeophycus*, *Scoyenia*) and unassigned vertebrate and invertebrate traces [30]. The SGDS also preserves a large collection of *Characichnos* swim tracks produced by theropods [31], [32]. *Grallator* tracks comprise approximately 95% of all dinosaurian footprints from all track-bearing horizons combined. In addition to its ichnofauna, Whitmore Point Member sediments in the St. George region have produced a diverse body fossil biota, including plant megafossils [33], ostracodes [34], conchostracans [35], fishes (hybodont sharks, coelacanths, lungfish, semionotids, and palaeoniscoids) [36], and fragmentary, as-yet unstudied theropod dinosaur elements.

## Results

### Ichnotaxonomy

*Eubrontes* Hitchcock, 1845 [37]

Figure 6a

**Figure 6:**
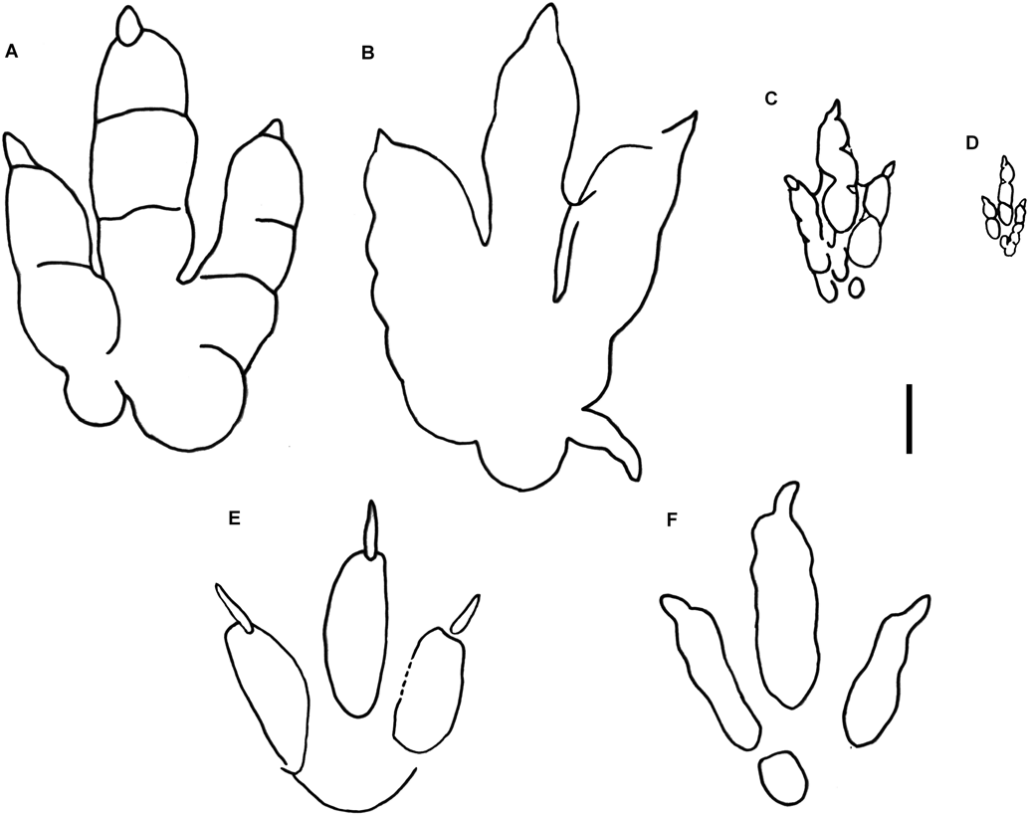
Schematic diagrams of Late Triassic-Early Jurassic theropod tracks. A, *Eubrontes*, referred specimen, right pes (AC 45/1; traced from [38]). B, *Gigandipus*, holotype, left pes (AC 9/16; traced from [47]). C, *Anchisauripus*, holotype, left pes (AC 4/6; traced from [38]). D, *Grallator*, holotype, left pes (reversed image of natural cast) (AC 4/1a; traced from [38]). E, *Dilophosauripus*, holotype, ?left pes (UCMP 79690; traced from [50]). F, *Kayentapus*, right pes from holotype trackway (UCMP 83668; traced and modified from [50]). Scale bar equals 5 cm. AC = Appleton Cabinet, Amherst College, Amherst, Massachusetts, United States of America; UCMP = University of California Museum of Paleontology, Berkeley, California, United States of America.

#### Diagnosis

*Eubrontes giganteus* has broad pes tracks >25 cm long, functionally tridactyl with short digit III, and divarication angles between 25°–40° [38].

#### Discussion

The ichnotaxonomy of Late Triassic and Early Jurassic tracks attributed to basal theropods includes a degree of subjectivity. Because large-bodied (>3 m), Early Jurassic theropods plesiomorphically retain fairly similar, unspecialized feet (compared to later Jurassic and Cretaceous taxa), multiple taxa are almost certainly represented within this one ichnotaxon. Tracks in the ambulatory portion of the SGDS.18.T1 trackway (Table 1) exhibit characteristics of *Eubrontes* [38] (diagnosed above). Because of the different posture adopted while crouching, the pes prints of the resting trace itself are somewhat different [39], [40].

Large, *Eubrontes*-like tracks from other Upper Triassic-Lower Jurassic formations, which are typically bipedal, tridactyl, and mesaxonic, have been considered distinct at the ichnogeneric level as *Anchisauripus*, *Dilophosauripus*, *Gigandipus*, and *Kayentapus* based on size and, to a lesser degree, morphological differences [38], [41]. These ichnotaxonomic distinctions have been questioned; at issue is whether ichnite morphology correlates more with actual taxonomic diversity or with variations in track maker-substrate interaction, and thus better represents paleoenvironment and behavior than taxonomy. Below, we compare and cite current criteria for the recognition of each ichnotaxon.

*Gigandipus* Hitchcock, 1856 [42]

Figure 6b

#### Diagnosis

Same as *Eubrontes giganteus* except including a medially or caudomedially oriented hallux impression [3], [4], [43]. Tail drag marks are present in the holotype and several referred specimens and has been touted as diagnostic [3], [44].

#### Discussion

*Gigandipus caudatus* tracks are similar to *Anchisauripus* and *Eubrontes* tracks except they invariably exhibit an impression of a medially or caudomedially oriented hallux. In various pedal proportions, *Gigandipus* is indistinguishable from tracks otherwise assigned to *Anchisauripus* and *Eubrontes*, so the ichnotaxon is reliably distinguished only by the presence of the hallux impression. There has been some speculation that *Gigandipus* is an extramorphological variant of *Eubrontes* in which the track maker's foot sank deep enough into the substrate to bring the normally elevated hallux into contact with the substrate [43], [45], but some *Eubrontes* tracks that lack hallux impressions are apparently deeper than some *Gigandipus* tracks [46], so foot-substrate interactions cannot universally explain these differences. Discrete intrataxonomic behaviors may also explain differences between *Gigandipus* and *Eubrontes*; in some ichnologic schemes (e.g., one where ichnotaxa are based entirely on quantitative and morphological criteria and behavioral differences are excluded), these would render the two synonymous [44]. *Gigandipus* tracks, with hallux impressions, are represented at the SGDS, suggesting that, at least locally, they may in fact be the result of foot-substrate interaction rather than two taxonomically distinct track makers. Several of the tracks in the progression away from the SGDS.18.T1 resting trace include hallux impressions, and could be assigned to *Gigandipus* were they viewed in isolation, but others do not. This supports the oft-hypothesized perception of *Gigandipus* as an extramorphological variant of *Eubrontes* and that, in at least some instances, the two ichnotaxa are synonymous. The SGDS.18.T1 trackway also possesses periodic tail drag marks associated with typical *Eubrontes* morphotype tracks.

*Anchisauripus* Lull, 1904 [47]

Figure 6c

#### Diagnosis

Tracks narrower than *Eubrontes* but broader than *Grallator*, 15–25 cm in length, divarication angles of outer digits 20°–35°, and digit III projection ratio between 1.3 and 1.8 (more than *Eubrontes* but less than *Grallator*) [38].

#### Discussion

The history of the ichnogenus *Anchisauripus*, and specimens referred to it, is especially convolute. In general, it has historically been a “wastebasket” for tracks that were larger than the accepted norm of *Grallator* (Figure 6d) but smaller than the accepted norm of *Eubrontes*. Indeed, even most modern usages depend heavily on size as a diagnostic criterion [38], though there do seem to be distinct proportion-based groupings of some ichnospecies [38], [46]. It has also been hypothesized that *Grallator*, *Anchisauripus*, and *Eubrontes* may (at least in some instances) represent an ontogenetic series, with attendant heterochronic morphological changes, of one or more theropod taxa [38]. In an older review of the ichnotaxon [4], *Anchisauripus* was thought to differ from either *Grallator* or *Eubrontes* by possessing a short, caudally-directed hallux impression that was frequently detached from the remainder of the print [43], but this has been interpreted (based on a specimen misidentified as the holotype [38]) as a fragment of a mud crack that intersects the impression [46]. However, in modern bird tracks, digit impressions, including the hallux, have been known to precipitate mud cracks [48], [49], so it remains to be seen whether or not *Anchisauripus* truly does possess a hallux impression. Many tracks at the SGDS fall within the *Anchisauripus* size range, but no morphological differences can be distinguished between them and smaller *Grallator* tracks, and, in the upper size range, *Eubrontes*.

*Dilophosauripus* Welles, 1971 [50]

Figure 6e

#### Diagnosis

None current (see Discussion, below).

#### Discussion

*Dilophosauripus williamsi* was first named for theropod tracks from the Kayenta Formation of northern Arizona [50] and are therefore geographically similar to, and only slightly younger than, the SGDS tracks. The only other report of this ichnotaxon was from Lower Jurassic strata in France [51]. It was originally differentiated from similarly-sized *Eubrontes* tracks largely by its possession of particularly long claw marks [50], [52], but these may be artifactual claw drag marks rather than reflective of a genuinely distinct morphology of the track maker's foot (per J. Farlow [52]). Its distinctiveness from *Eubrontes* and/or *Kayentapus* is therefore suspect pending further investigation.

*Kayentapus* Welles, 1971 [50]

Figure 6f

#### Diagnosis

The ichnogenoholotypic trackway of *Kayentapus* (for *K. hopii*) demonstrates significant variation from print to print [52], making a morphological diagnosis for the taxon difficult to establish, but the ichnogenus may be characterized by slender digits that taper less and have less acute angles of divarication than those of either *Grallator* or *Eubrontes* [1], [53]. A more stringent, quantitative diagnosis also includes: length between 11.5–40 cm, metatarsophalangeal pad of digit IV well defined, and angle of divarication between digits III and IV greater than that between digits II and III [41].

#### Discussion

The ichnogenotype, *Kayentapus hopii*, was named at the same time, and for tracks in the same area, as *Dilophosauripus* [50]; other ichnospecies have also been referred to the ichnogenus [46], [54]. It may be synonymous with the previously named *Apatichnus* and/or *Talmontopus* [1], [41] and later named *Schizograllator* and *Zizhongpus* [5], [53]; several ichnotaxa erected based on specimens from southern Africa [55] may also be synonymous [41]. Like *Anchisauripus*, *Kayentapus* has been differentiated from *Grallator* and *Eubrontes* almost exclusively on the basis of its intermediate size between *Grallator* and *Eubrontes* [1], [38], [46], [52]; recently discovered specimens from the SGDS differ only slightly in size from *Eubrontes* tracks at the same locality. However, the validity of *Kayentapus* has been upheld based on differences in the degree of digit III projection and degree of divarication of digit IV; *K. minor* and *K. soltykovensis* plot apart from other ichnotaxa in proportions involving print length, width, and the degree to which digit III projects beyond other digit impressions [46], [53].

### Description

The beginning of the SGDS.18.T1 trackway has a southerly orientation, approximately parallel to the paleoshoreline trend. The track maker first proceeded up the stoss side of an erosive mega-ripple (berm) with an approximately 10° slope and then stopped, placing both feet parallel. It then lowered its body, bringing the metatarsals and ischial callosity into contact with the substrate, creating nearly symmetrical, elongate “heel” and circular ischial impressions (Figures 4, 5, Table 2.) These are similar to previously described *Eubrontes* and *Grallator* traces [5], [7], [8]. The absence of a broad, linear impression immediately caudal to the ischial callosity trace indicates that even while seated, the *Eubrontes* track maker kept the proximal portion of its tail elevated. A tail mark 31 cm in length and located 134 cm caudal to the ischial callosity but aligned with the crouching trace axis indicates that the distal tail made substrate contact.

**Table 2:** Measurements (in cm; ° as noted) of SGDS.18.T1 *Eubrontes* resting trace and immediately associated marks.

Track #	L/R	M/P	TL	TW	MTD	TTL	I–II	I–III	I–IV	II–III	II–IV	III–IV
TD1	n/a	n/a	31	1.7	(1.5)	n/a	n/a	n/a	n/a	n/a	n/a	n/a
Isch 1	n/a	n/a	10.5	(9.5)	–	n/a	n/a	n/a	n/a	n/a	n/a	n/a
Isch 2	n/a	n/a	(11)	(10)	–	n/a	n/a	n/a	n/a	n/a	n/a	n/a
T1.4	R	P	(34)	(20)	(4)	(56)	–	–	–	21°	56°	32°
T1.5	L	P	?35	(24)	3.5	(46)	–	–	–	37°	(78°)	(39°)
T1.6	R	P	(31)	(20)	(2)	(42)	–	–	–	33°	65°	28°
T1.7	L	P	(35)	(24)	3.5	(53)	127°	148°	174°	22°	55°	34°
T1.8	L	M	(22)	(12)	(2)	n/a	n/a	n/a	n/a	n/a	n/a	n/a
T1.9	R	M	(18)	(8.5)	(2)	n/a	n/a	n/a	n/a	n/a	n/a	n/a
Cranial Isch 1 to cranial Isch 2					(25)							
Cranial R P 1 digit IV to cranial R P 2 digit IV					(21)							
Cranial L P 1 digit IV to cranial L P 2 digit IV					(24)							
Medial L M to medial R M					55							
Medial 1^st^ L P to medial 1^st^ R P					(21)							
Medial 2^nd^ L P to medial 2^nd^ R P					(23)							
Exterior L M to exterior R M					73							
Exterior 1^st^ L P to exterior 1^st^ R P					(65)							
Exterior 2^nd^ L P to exterior 2^nd^ R P					(56)							
Cranial end of TD1 to caudal end of Isch 1					134							
Caudal end of TD1 to caudal end of Isch 1					165							

Roman numerals = divarication angles between indicated pedal digits, Isch = ischial callosity impression, L/R = left or right, M/P = manus or pes, MTD = maximum trace depth, TD = tail drag, TL = total trace length (excluding metapodial impression, if any), TTL = total trace length (including metapodial impressions, if any), TW = total trace width. n/a = measurement not applicable, – = measurement not obtainable.

Measurements in parentheses are approximations due to incompleteness, poor preservation of trace, or ambiguity in discerning track margin from surface sedimentary structure.

Unlike in any other known resting theropod trace, its position on a slope enabled the SGDS.18.T1 track maker to bring both hands into contact with the substrate a short distance craniolateral to the pes impressions. The manus impressions are unique, exhibiting medially-directed digits unlike any previously seen in an ichnite attributable to a theropod.

After resting in this position, the animal shuffled forward about 25 cm and paused once again, leaving new pes, metatarsal, and ischial, but not manual, impressions. The new right pes impression overprinted the caudal right manus impression, and the claw on left pedal digit II registered a drag mark from the first resting position to the second. After an indeterminate amount of time, the theropod then stood and proceeded forward, left pes first. Once fully erect, the track maker walked across the remainder of the exposed surface at speeds (determined by stride lengths) that vary with the undulating topography, leaving intermittent, thin, linear, and nearly sagittal drag marks from the distal end of the tail (Figures 4, 5). The majority of digit I (hallux) traces in the remainder of the trackway can be seen only in the left footprints (Table 1).

## Discussion

The medially-directed digit impressions on the manus traces strongly support avian-style anatomical restrictions in the mobility of the theropod wrist. Traditionally, theropod hands have been reconstructed with palms facing ventrally, possibly in adherence to the plesiomorphic tetrapod state retained in crurotarsan archosaurs [56], [57] and in contrast to the palms-medial (semi-supinated) condition seen in the adducted thoracic limbs of extant, avian theropods [58], [59]. Recent functional analyses of theropod thoracic limbs from the Late Jurassic through the Late Cretaceous, however, indicate that non-avian theropod arms were unable to pronate/supinate, implying that the manus could only articulate in line with the radius and ulna [58], [60] such that the palms faced medially, not ventrally, and the digital sequence (I–III, IV, or V) proceeded from dorsal to ventral rather than medial to lateral. This is the configuration present in birds when the forelimbs are adducted. Although bipedal theropods would rarely have made manus prints, ichnology provides a means of testing whether or not very early theropods, for which wrist mobility is unknown, also conformed to this pattern.

**Figure 7:**
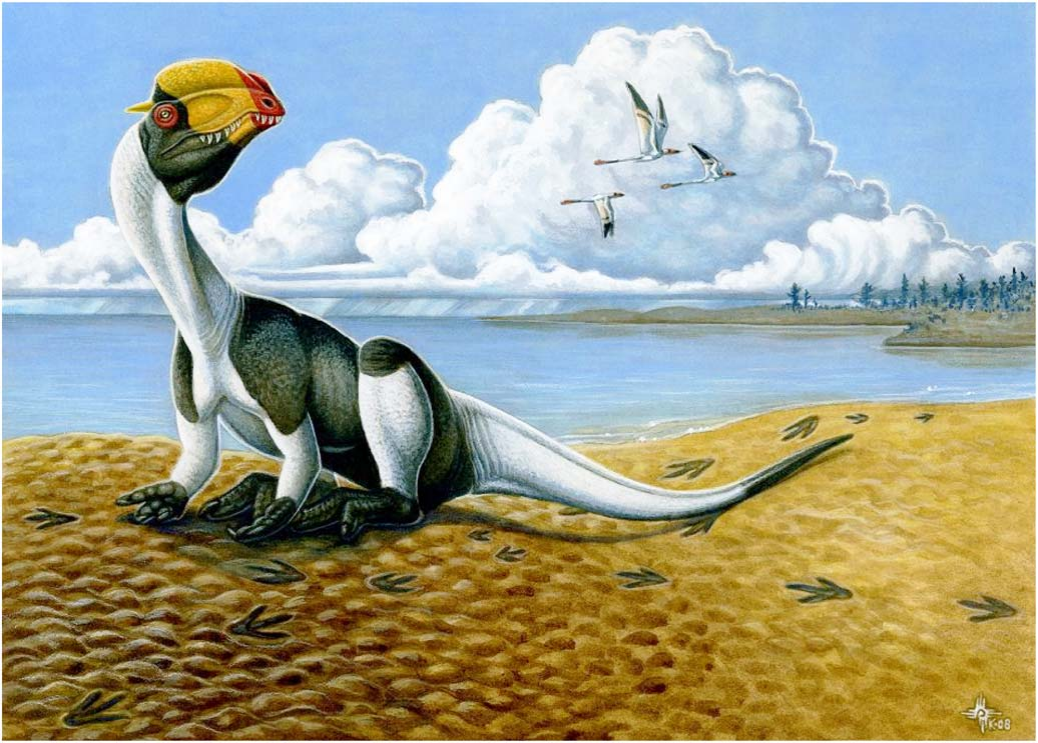
Restoration of Early Jurassic environment preserved at the SGDS, with the theropod *Dilophosaurus wetherilli* in bird-like resting pose, demonstrating the manufacture of SGDS.18.T1 resting trace. By Heather Kyoht Luterman.

The only theropod body fossils thus far reported from the Moenave Formation were attributed to the coelophysoid *Megapnosaurus* sp. [61], which is too small to have made *Eubrontes* tracks. The larger *Dilophosaurus wetherilli* from the overlying (and therefore slightly younger) Kayenta Formation in Arizona [62], which is either a coelophysoid [63] or a slightly more derived basal neotheropod [64], is of appropriate size and a suitable model for the SGDS.18.T1 track maker (Figure 7), though the existence of *Dilophosaurus* itself during Moenave time is not indicated. Coelophysoids, possibly including *Dilophosaurus*, are the most basal definite theropods known; a few, more basal dinosaurs, such as herrerasaurids and *Eoraptor*, may [65] or may not [66] be theropods [67]. Such basal taxa are unknown from Jurassic strata and thus are not parsimoniously potential SGDS.18.T1 track makers.

Although it is possible that the SGDS.18.T1 manus impressions involve some movement of the appendages during registration, they clearly exhibit impressions of at least two ungual-bearing digits. The manus of basal, Early Jurassic theropods, such as coelophysoids [62], [68], bear unguals only on digits I–III; a non-ungual phalanx terminates digit IV. If a manus with the avian-style configuration was brought straight down in standard theropod resting posture [60], only the ventral (lateral) surface of the outermost digit would contact the substrate, and only one narrow digit impression would be discernible; all other (“inner”) digits would rest atop the outermost and not make discrete impressions. In order to impress multiple digits from a crouching posture, the arms must have been flexed at the elbow, and possibly the wrist, approaching the inclined substrate at an acute angle such that the dorsolateral surfaces of several differentially flexed, outermost digits made contact (Figures 4, 5). The impression of diminutive digit IV, if indeed this digit was not embedded wholly within the palmar region, is indistinct within the larger overall impression. Neither the belly nor the elevated, more proximal portions of each forelimb created impressions. The arms must therefore have been extended from the body, rather than the entire body leaning forward far enough to bring arms in neutral resting posture into substrate contact (Figure 7). The medially, not cranially, oriented manual digits indicate that even while resting, the track maker was incapable of supinating its hands to create palms-down impressions, as suggested by anatomical studies of geologically much younger theropods.

Several other manus- and pes-bearing tracks of Late Triassic-Middle Jurassic ichnotaxa have been attributed to quadrupedal theropods. In these specimens, hand print digital formulae and proportions reportedly match the manus morphologies of contemporaneous basal, coelophysoid theropods [62], [68], [69]. But when discernible at all, these specimens exhibit forward-pointing digit impressions. Such prints could only be manufactured by hands with either fully pronated (or supinated) orientations, anatomical impossibilities in more recent theropod osteological reconstructions [58], [60]. A brief review of these ichnotaxa is therefore warranted: if correctly attributed to theropods, their greater numbers suggest that SGDS.18.T1 is somehow anomalous, either pertaining to a group of theropods that possessed a different forelimb morphology, or not made by theropods. It would also imply that the medially-facing manus configuration is characteristic of, and evolved in, a smaller, less inclusive group of more derived theropods.

*Agialopous* Branson and Mehl, 1932 [70]

*Agialopous wyomingensis* Branson and Mehl, 1932 [70]

The now-lost holotype specimens of the ichnite *Agialopous wyomingensis*, from the Upper Triassic Bell Springs Formation of Wyoming, ostensibly included a pair of purported manus and pes prints [70]. Based on pes print morphology, *Agialopous* is likely a junior synonym of *Grallator* [71]. The supposed manus print appears to be a smaller pes print preserved somewhat differently from that of the larger, main track and thus does not constitute a genuine manus impression [71].

*Atreipus* Olsen and Baird, 1986 [6]

*Atreipus* ispp.

Thorough reviews of this controversial ichnogenus and its multiple ichnospecies have previously been published [6], [46], [69], [72], but it continues to vacillate between assignments to theropodan or ornithischian track makers. The ichnotaxon is universally quadrupedal, possessing both manus and pes prints. As far as is currently known, it is also exclusively Late Triassic [6]. The pes prints are extraordinarily similar to those of *Grallator*, and indeed many examples of *Atreipus* have, at one time or another, been referred either to *Grallator* or other similar theropod ichnotaxa (e.g., *Anchisauripus*). *Atreipus* has typically been considered evidence that at least some early theropods were at least facultatively quadrupedal [43], [46], [69], [73]–[76]. The highly digitigrade manus prints of some ichnospecies are tridactyl; others are tetradactyl; in many ways, both the pes and especially manus prints resemble those of the non-dinosaurian, chirotherian ichnotaxa [6], [72]. In all ichnospecies, the manual digit impressions face cranially, roughly paralleling the pedal digits. In most ichnospecies, the manus impressions include marks made by small claws, even on the impression of digit IV. The small claw size has led some [6], [7], [77]–[79] to settle on a track maker that was either an early, non-saurischian and non-ornithischian dinosaur, or a *bona fide* ornithischian, albeit one with no known skeletal correlate. A third interpretation of *Atreipus* as made by a non-dinosaurian dinosauriform [80] has also been proposed [72]. As noted above, the functional morphology of the theropod forelimb [58], [81] makes assignment of *Atreipus* to theropods unlikely.

*Banisterobates* Fraser and Olsen, 1996 [82]

*Banisterobates boisseaui* Fraser and Olsen, 1996 [82]

The holotype of *Banisterobates boisseaui* is a single natural cast of an unusually small trackway consisting of three pes and two manus prints from the Upper Triassic Dry Fork Formation (Newark Supergroup) of Virginia. They have proven difficult to attribute to any higher taxon [82]. The track maker appears to have had a functionally tetradactyl pes with a short, cranially-oriented hallux; the tracks also exhibit faint “heel” impressions. The manus prints appear to be tridactyl, with forward-pointing digits, but lack distinct claw impressions. Its describers [82] ruled out non-dinosauriform archosaurs, but the tracks are morphologically consistent with either non-dinosaurian dinosauriforms (e.g., *Marasuchus* [83]), basal theropods, or basal ornithischians. They preferred an ornithischian interpretation based on the forward-pointing hallux and presence of manus impressions.

*Changpeipus* Young, 1960 [84]

*Changpeipus carbonicus* Young, 1960 [84]

Closely associated with one of several, apparently isolated, tridactyl theropod tracks named *Changpeipus carbonicus* from the ?Middle Jurassic of Liaoning, China, was a tiny tridactyl print that was interpreted as a manus impression of a theropod, although pertaining to a different individual than the pes print maker [84]. There is, however, no reason to assume that this is correct: in isolation, it appears to be a small pes print of a theropod (whether or not the same ichnotaxon) [69], and its position lateral to the nearest similarly-oriented pes print translates into a bizarre, untenable posture for any known theropod. It does not represent a theropod manus impression.

*Delatorrichnus* Casamiquela, 1964 [85]

*Delatorrichnus goyenechei* Casamiquela, 1964 [85]

When first described [85], *Delatorrichnus goyenechei* was considered to have been made by a theropod progressing quadrupedally. Few other tracks have been referred to this *Atreipus*-like ichnotaxon [79], but include some from the Kayenta Formation of southeastern Utah that lack manus prints [86]. Like *Banisterobates*, *Delatorrichnus* tracks are quite small and possibly represent juveniles of a larger taxon [86]. *Delatorrichnus* manus prints are only slightly smaller than, and lie immediately adjacent to, their associated pes prints. Like *Atreipus* and *Banisterobates*, the digits of the manus prints are oriented cranially, diverging only slightly from the axes of the pedes [85], and thus are unlikely to represent theropods.

*Kayentapus* Welles, 1971 [50]

*Kayentapus minor* Weems, 1992 [46]

A small percentage of a large number of tracks assigned to *Kayentapus minor* from the Upper Triassic Groveton Member of the Bull Run Formation (Chatham Group, Newark Supergroup) were reportedly accompanied by largely amorphous, ovoid impressions that have been interpreted as manus impressions [10] based on their relative positions with respect to their associated pes prints, although the positions of the “manus” impressions were inconsistent between specimens. Similar shapeless impressions were reported near a track of *Eubrontes* in the roughly coeval East Berlin Formation of Connecticut [9] and with a crouching *Grallator* specimen from the Navajo Sandstone at Coyote Buttes in south-central Utah [8]. Weems [10] interpreted the “manus” impressions of *Kayentapus* as made by theropods that hyperextended their manual digits when dropping into a quadrupedal stance (a posture also used to explain the morphology of the manus impressions associated with the crouching *Grallator* trace [8]). Tacitly, this interpretation also applies to the Connecticut *Eubrontes* track and Coyote Buttes *Grallator* specimen, as well as tracks of *Atreipus* and *Banisterobates* and tracks typically assigned to “prosauropods,” such as *Navahopus* and *Otozoum*. This interpretation posits that manual digit impressions are absent because the digits were never impressed in the first place; the amorphous impressions represent palm-only impressions. This hypothesis was supported by observations that the manual phalanges of coelophysoid theropods (the most likely track makers) exhibit proximodorsally extended distal articular surfaces that permitted digit hyperextension [62]. Weems [10] and others [60] have argued that this ability in theropods enhanced a raptorial function of the manual digits during predation, but Weems actually ascribed such ability and behavior to *all* Late Triassic-Early Jurassic saurischians, including basal sauropodomorphs (“prosauropods,” specifically *Massospondylus*), which are not typically perceived as predators; the need for this ability in those taxa was not explained.

We accept the hyperextensive ability in the studied theropod taxa, but challenge the adaptive scenario supporting it [10]: to prevent the manual claws from becoming dull with repeated contact with the sediment. This seems unsatisfactory for several reasons:

1. It does not adequately explain why this ability was absent in later theropods, especially many maniraptorans, such as deinonychosaurs, for which the arms have generally been ascribed a raptorial function. It is additionally peculiar because Cretaceous examples of ostensible theropod claw marks are known [87].
2. The regenerative ability of keratinous ungual sheaths were almost certainly sufficient to heal any damage occasional contact with coarse sediment may have inflicted. The “prosauropod” tracks Weems discusses provide a good analogy: at least some basal sauropodomorphs (such as *Melanorosaurus*) were probably facultatively quadrupedal [88] and had even larger manual claws than contemporaneous theropods. *Melanorosaurus*, and perhaps other taxa, re-evolved an at least semi-pronated manus as an adaptation for propulsive forelimb motion during quadrupedality [88]. Tracks possibly made by quadrupedal basal sauropodomorphs, such as *Navahopus* (an alternative affiliation of the *Navahopus* track maker has been proposed [89]), show that the track makers regularly placed these claws into a variety of sedimentary substrates, including coarse, quartzose sand [90], [91]. Regular contact with any substrate, and coarse sand in particular, would have worn down the keratinous sheaths of the claws much faster than would have occasional contact with the fine mud in which tracks are typically preserved, yet the *Navahopus* track maker either lacked or did not utilize an ability to hyperextend the manual digits to keep them from contacting the substrate – indeed, the holotype of *Navahopus* [90] represents an animal climbing a dune face of loose sand, wherein use of the claws to find additional purchase would be useful.
  Weems [10] specifically stated that the theropod makers of the *Kayentapus* prints (as well as *Atreipus* and *Banisterobates*, for which he accepted a theropod track maker) only occasionally adopted a quadrupedal stance, and then only when resting – not for prolonged locomotion. At rest (i.e., with little or no movement), manual claws could have entered the finer-grained, less abrasive substrate with little potential for wear. Moreover, many later theropods, particularly some Jurassic and Cretaceous maniraptorans, have been interpreted as arboreal [92], [93]–[95], demonstrating a need to actively use claws (including manual unguals) to aid in climbing – in short, to regularly and readily place their claw tips in contact with rough, abrasive surfaces (tree trunks and branches). If the capacity of theropods to rapidly regenerate their keratinous unguals was insufficient to permit occasional resting in contact with mildly abrasive sediment, then it certainly was insufficient for climbing or even raptorial functions.
3. As noted above, the ability of a theropod to make a palm-only manus impression is contraindicated by functional studies: the inability to pronate/supinate the distal forelimb would make it impossible for the manus to be oriented in such a way that the palmar surface of the manus could be brought into contact with the substrate. The long axes of the ovoid “manus” impressions from Virginia [10] and Utah [8] are oriented approximately parallel to the long axes of their pes prints. As ostensible impressions made by adjacent distal metacarpals, this means that the digits of the manus that made this print would have to be oriented either strongly outward or strongly inward – in either case, almost perpendicular to the orientation of the pedal digits, in marked contrast to the directions of the manual digits in other ostensible theropod manus prints (e.g., *Atreipus* and *Banisterobates*) and in anatomically unfeasible positions.

Invoking hyperextension of the manual digits when adopting a quadrupedal stance seems wholly unnecessary, and we doubt whether this happened regularly. Thus, it is impossible to verify whether or not any of the shapeless impressions accompanying the Culpeper *Kayentapus* or Coyote Buttes *Grallator* prints actually are manus impressions. It is possible that these impressions represent not the palmar but the ventral (lateral, or outermost) or dorsal surface of the manus and/or digits, made in a fashion similar to that described here for SGDS.18.T1 and in agreement with the understood function of the theropod forelimb. Unlike SGDS.18.T1, however, multiple, distinct digits did not leave impressions.

*Masitisisauropus* Ellenberger, 1972 [55]

*Masitisisauropus palmipes* Ellenberger, 1972 [55]

Tracks assigned to *Masitisisauropus palmipes* were initially thought to represent manus and pes prints of a possibly feathered, Late Triassic bird or bird-like non-avian theropod [96]. The purported feather impressions are suspect [97]; *Masitisisauropus* may be synonymous with *Grallator* [76], [98], but the association of the purported manus prints with the pes prints has not yet been reinvestigated, and the possibility remains that, like *Agialopous*, the manus and pes prints represent unrelated pes tracks of different individuals.

Other Tracks

A poorly preserved, Early Cretaceous trackway from England has been interpreted as a trace of a large, quadrupedal “carnosaur” (referred to the “wastebasket” taxon *Megalosaurus*) [69], [98], [99]. The poor preservation of these tracks, and their association with more common *Iguanodon*-type footprints, many of which were made by quadrupeds, has led to doubt about the correct affinity of these tracks.

Other accounts of possible theropod paired manus and pes prints are either poorly preserved [9], consist of tracks of multiple taxa in close proximity, or demonstrably pertain to ornithischians [5], [69].

### Conclusions

In summary, other ostensible theropod manus prints are either dubiously attributable to theropods, dubiously made by the manus of a pes-print maker, or uninformative with regard to the track maker's forelimb functional morphology. Because the crouching traces in the trackway SGDS.18.T1 match the architecture of known theropods, we support the alternative interpretation that most, if not all, other prints showing manus impressions instead pertain to ornithischian or other non-theropodan dinosaurs or dinosauriforms [6] with functionally tridactyl pedes. SGDS.18.T1 therefore includes the only unambiguous theropod manus impressions recognized to date and indicates that the avian orientation of the manus, with medially-facing palms, evolved very early within the Theropoda. Less parsimoniously, this posture evolved in immediate dinosaur ancestors; absence in other dinosaurs would thus constitute reversals.

The lack of marks in SGDS.18.T1 made by the distal thoracic and pelvic limbs and the ventral portion of the pelvis indicate that, while resting, even the earliest theropods adopted a modern ratite-like [100] posture (Figure 7) with the legs folded symmetrically beneath the body such that the weight of the body was distributed between each metatarsus and pes. The oldest known body fossil evidence for adoption of this posture in a theropod is preserved in Late Cretaceous oviraptorosaurians [101] and two Early Cretaceous troodontids [102], [103]. Except in a specimen from the Navajo Sandstone at Coyote Buttes, Arizona [8], the metatarsal and pes impressions of *Grallator* and other theropod resting traces exhibit ambiguous symmetry [5], [7]. The clear symmetry of SGDS.18.T1 demonstrates that even some of the oldest, basal-most theropods engaged in this additional avian-style behavior, which therefore also evolved very early in the theropod lineage or was retained in theropods from pre-dinosaurian archosaurs.

## Materials and Methods

Latex peels of the SGDS.18.T1 crouching trace are also reposited at the SGDS and at the University of Colorado at Denver Dinosaur Tracks Museum (UCD) as UCD 177.77. Measurements were made using a square-meter grid with 10 cm partitions and a Brunton compass, and from tracings of the ichnites (reposited as UCD T 472 and T 642) using tape measures and protractors. Photogrammetry (Figure 4) utilized an Olympus C8080 Wide Zoom digital camera mounted on a tripod equipped with a right-angle extension arm that allowed the camera to be positioned perpendicular to the track surface to minimize distortions. The stereoscopic images used a ground sample distance of 0.6 mm and were processed using ADAM Technology 3D Analyst, resulting in a 3D digital terrain surface and orthorectified images [104]. Comparative analysis involved examination of original materials and published descriptions.
